# Effectiveness of a Smartphone-Based Stress Management Program for Depression in Hospital Nurses During COVID-19 in Vietnam and Thailand: 2-Arm Parallel-Group Randomized Controlled Trial

**DOI:** 10.2196/50071

**Published:** 2024-08-30

**Authors:** Kazuhiro Watanabe, Thuy Thi Thu Tran, Narisara Sripo, Asuka Sakuraya, Kotaro Imamura, Plernpit Boonyamalik, Natsu Sasaki, Thanate Tienthong, Hiroki Asaoka, Mako Iida, Quynh Thuy Nguyen, Nga Thi Nguyen, Son Thai Vu, Thuy Thi Ngo, Tham Thi Luyen, Long Duc Nguyen, Nga Thi Viet Nguyen, Binh Thanh Nguyen, Yutaka Matsuyama, Yukie Takemura, Daisuke Nishi, Akizumi Tsutsumi, Huong Thanh Nguyen, Orawan Kaewboonchoo, Norito Kawakami

**Affiliations:** 1 Department of Public Health Kitasato University School of Medicine Sagamihara Japan; 2 Faculty of Environmental and Occupational Health Hanoi University of Public Health Hanoi Vietnam; 3 Princess Agrarajakumari College of Nursing Chulabhorn Royal Academy Bangkok Thailand; 4 Department of Digital Mental Health Graduate School of Medicine The University of Tokyo Tokyo Japan; 5 Faculty of Public Health Mahidol University Bangkok Thailand; 6 Department of Mental Health Graduate School of Medicine The University of Tokyo Tokyo Japan; 7 Faculty of Social Science and Behavior Hanoi University of Public Health Hanoi Vietnam; 8 Pho Noi Hospital Hung Yen Province Vietnam; 9 Xanh Pon Hospital Ha Noi Vietnam; 10 Department of Biostatistics School of Public Health Graduate School of Medicine, The University of Tokyo Tokyo Japan; 11 Nursing Department University of Tokyo Hospital Tokyo Japan

**Keywords:** digital mental health intervention, unguided program, universal prevention, health care workers, nurses, COVID-19, depression, mobile phone

## Abstract

**Background:**

During the COVID-19 pandemic, health care professionals experienced high levels of depression. However, extant research has not highlighted effective internet-based psychological interventions to improve the mental health in this population during the pandemic. It remains unclear whether self-guided, internet-based cognitive behavioral therapy (iCBT) programs are effective in improving the mental health of health care workers during the COVID-19 pandemic.

**Objective:**

The aim of this study was to evaluate the effectiveness of a smartphone-based iCBT stress management program for reducing the depression experienced by nurses in Vietnam and Thailand.

**Methods:**

From March to April 2022, a 2-arm, parallel-group randomized controlled trial was implemented. One arm offered a 7-week self-guided iCBT program, and the other offered treatment as usual as a control arm. Full-time nurses were recruited from 6 hospitals: 2 hospitals in Vietnam and 4 hospitals in Thailand. The primary outcome of this program was the severity of depression measured by the Depression Anxiety Stress Scale-21 items. Follow-up surveys were conducted to measure the change in depression severity at 3 months (July-August 2022) and at 6 months (October-November 2022) after baseline. Mixed modeling for repeated measures was used to test the effects of the intervention compared with the control for the follow-up.

**Results:**

A total of 1203 nurses were included in this study: 602 in the intervention group and 601 in the control group. The follow-up rate at 3 and 6 months ranged from 85.7% (515/601) to 87.5% (527/602). The completion rate for the program was 68.1% (410/602). The group difference in depression was significant at the 3-month follow-up (coefficient=–0.92, 95% CI –1.66 to –0.18; *P*=.02) and nonsignificant at the 6-month follow-up (coefficient=–0.33, 95% CI –1.11 to 0.45; *P*=.41). The estimated effect sizes were –0.15 and –0.06 at the 3- and 6-month follow-ups, respectively.

**Conclusions:**

Our study shows that the smartphone-based iCBT program was effective in reducing depression at the 3-month follow-up among hospital nurses in Vietnam and Thailand during the COVID-19 pandemic. However, the effect size was small, and therefore, these results may not be clinically meaningful.

**Trial Registration:**

UMIN Clinical Trials Registry UMIN000044145; https://center6.umin.ac.jp/cgi-open-bin/ctr/ctr_view.cgi?recptno=R000050128

**International Registered Report Identifier (IRRID):**

RR2-10.20944/preprints202303.0450.v1

## Introduction

### Background

The COVID-19 pandemic started in late December 2019 and is still impacting the physical and mental health of people worldwide [1]. Health care workers were the most affected during the COVID-19 pandemic, as they experienced increased workload while providing care for patients, along with anxiety about the high risk of infection from exposure to COVID-19 and dealing with stigmatization and discrimination from the public at large. During the COVID-19 pandemic, health care professionals experienced high levels of depression, anxiety, and posttraumatic stress symptoms [2]. In particular, nurses experienced poorer mental health than other health care professionals [2]. A universal approach is important for improving the mental health of nurses, including that of the healthy nurses, given that every nurse is at risk of their mental health being negatively affected by COVID-19. Improving their mental health will make the quality of their work life better and ensure that the workforce provides appropriate health care by preventing their mental health issues from taking a toll on them.

Very few studies have highlighted effective interventions that can improve the mental health of health care workers during infectious disease epidemics such as SARS, Ebola, and Middle East respiratory syndrome [3]. During the Ebola epidemic, a training program on Psychological First Aid helped prevent burnout among primary health care workers in the community [4]. During the SARS outbreak, a comprehensive organizational intervention, including infection measures, protection training, and psychological support teams, for patients and health care workers reduced depression and improved sleep among nurses in a hospital [5]. A few studies have reported positive responses to group-based resilience training [6] and an improvement in self-efficacy and interpersonal problems after conducting a web-based resilience training program among health care workers during the influenza pandemic [7]. In addition, other types of psychological interventions have been found to be effective in improving the mental health of frontline health care workers and other responders: eye movement desensitization and reprocessing, trauma risk management [8], supportive psychotherapy [9], peer support programs [10], and mindfulness- and mediation-based interventions [11].

As close face-to-face personal contact was limited to prevent the spread of COVID-19 in an organization or community, using information and communication technology to provide training on stress management skills, such as internet stress management programs [12], has been encouraged [13]. Internet-based programs have additional strengths in accessibility, low cost, and confidentiality. These programs can be completed at convenient times, which is considered suitable for health care workers who work irregular schedules. A randomized controlled trial (RCT) reported that an internet-based cognitive behavioral therapy (iCBT) program reduced perceived work-related stress among nurses in the United States [14]. The effectiveness of non–face-to-face CBT interventions for improving the mental health of health care workers has been reported to be similar to that of face-to-face interventions [15]. We found that a self-guided iCBT program reduced depression among nurses in Vietnam during a 3-month follow-up [16].

However, these studies were conducted before the COVID-19 pandemic. In the early stages of the pandemic, e-mental health solutions were summarized to assist health care workers [17]. CBT was also delivered to nurses for insomnia as a digital sleep intervention, and its effect on insomnia was confirmed [18]. An RCT conducted in Spain during the COVID-19 pandemic reported that an internet-based stress management program focusing on psychoeducation and mindfulness skills training yielded no significant improvement in depression, anxiety, or stress among health care workers [19]. A recent RCT reported that a therapist-guided, 6-week, internet- and CBT-based stress recovery intervention for nurses improved stress recovery, anxiety, and depression symptoms [20]. However, it remains unclear whether self-guided iCBT programs are effective in improving the mental health of health care workers during the COVID-19 pandemic, where health care workers experienced far higher stress than usual.

### Objectives

Southeast Asia is one of the most populous geographical areas in the world, where rapid aging of the society is currently underway. The shortage of the health care workforce was serious in this region even before the COVID-19 pandemic [21]. As of the end of 2022, this region had the largest total mortality owing to COVID-19 followed by Europe and the United States [22]. Our study aims to evaluate the effectiveness of a 7-week smartphone iCBT stress management program for improving the mental health of nurses in this region during the COVID-19 pandemic by using a modified version of our previous iCBT program for nurses in Vietnam [16] adapted to suit the COVID-19 situation. To increase the generalizability of our findings, we selected 2 rapidly industrialized middle-income countries in the Southeast Asian region, namely, Vietnam and Thailand, to conduct the effectiveness study as part of the “Coping with COVID-19 helps Nurses be Active, Tough, and Smiling (COCONATS)” project. As the primary outcome, depression symptoms were measured. We hypothesized that depression scores in the intervention group would significantly improve when compared with those in the control group.

## Methods

### Trial Design

The trial design was an open, 2-arm, parallel-group RCT. Of the 2 arms, one offered the iCBT program for 7 weeks, and the other received occupational health and other services in their hospitals as a control group. After the completion of a web-based survey at baseline, nurses in each hospital were randomly assigned to the intervention or control groups at a 1:1 ratio. Randomization was conducted by stratifying the sample by countries, hospitals, and severity of depression at baseline. Follow-up surveys to measure the change in the outcome were conducted at 3 and 6 months after baseline. The web-based baseline and follow-up surveys were conducted independently in (not as a part of) the iCBT program. The study protocol was registered at the University Hospital Medical Information Network Clinical Trials Registry (UMIN000044145) and was published elsewhere [23]. This report of a randomized trial conforms to the guidelines in the CONSORT (Consolidated Standards of Reporting Trials) [24,25] (Multimedia Appendix 1).

### Study Setting

From March to April 2022, this RCT was conducted in 6 hospitals across Vietnam and Thailand. In Vietnam, 1 hospital in Hanoi and another province hospital in the north of Vietnam were selected as the study fields. After obtaining consent from the hospitals, lists comprising 723 full-time registered clinical nurses in the hospitals were sent to researchers at the Hanoi University of Public Health. These researchers sent the research project advertisement (flyers) and documents that explained the aim and procedure of the study, along with the baseline questionnaire (February to March 2022) to the potential participants. When the nurses agreed to participate, they signed an informed consent form and completed the baseline survey (March to April 2022). After the randomization, the intervention was provided to the intervention group for 10 weeks. The follow-up surveys were conducted at 3 months (July 2022) and 6 months (October 2022) after baseline.

In Thailand, 4 provincial hospitals at the tertiary level and above, located in Bangkok, Samut, Sakhon, and Lop Buri, joined this study. The principal investigator of Mahidol University set up a web-based meeting with local collaborators of the target hospitals to explain the research project and sent the advertisement to the collaborators who introduced the research project to employed nurses (N=4837). Later, the main research team invited all the potential participants to a web-based meeting to reread the aims and steps of the project (February to March 2022). In the meeting, the potential participants accessed and pressed accept on the web-based informed consent form to electronically sign it and begin completing the baseline questionnaire (March to April 2022). Those who were unable to attend the meeting could request project documents or ask for more information from the principal investigator or local collaborators during the application period. After the randomization, the intervention was provided to the intervention group for 10 weeks. The follow-up surveys were conducted at 3 (July to August 2022) and 6 months (October to November 2022) after baseline.

### Ethics Approval

The study protocol was approved by the research ethics review board of the Graduate School of Medicine/Faculty of Medicine, The University of Tokyo (32021082NI-1), Hanoi University of Public Health (353/2021/YTCC-HD3), and Mahidol University (MU MOU COA 2021-001). Informed consent was obtained from all the participants after full disclosure and explanation of the purpose and procedures of the study. The participants were informed that their participation was voluntary, that they could withdraw from the study at any time without stating reasons, and that nonparticipation and withdrawal would not result in any disadvantage to them.

### Recruitment

This RCT recruited nurses working in hospitals in Vietnam and Thailand. Nurses who were included were employed full-time as registered nurses at one of the 6 hospitals in Vietnam or Thailand and were able to access the internet via a mobile device such as a smartphone. The study excluded those who were (1) on sick or maternity leave at baseline or planned to take maternity leave or to leave or change their job in the next 6 months, (2) assistant nurses or helpers, (3) nonregular or part-time employees, (4) on sick leave for 10 or more days for a physical or mental condition in the past 4 weeks, and (5) receiving treatment for a mental health problem from any health care professional.

### Interventions

#### iCBT Program as the Intervention

The participants allocated to the intervention group were offered a 7-week smartphone-based self-guided iCBT program for nurses during the COVID-19 pandemic in Vietnam and Thailand. “The ABC Stress Management–COVID-19 version” was developed. This program, which consists of texts and static visuals of cartoon characters talking on the study topic and guiding participants through the learning (Figure S1 in Multimedia Appendix 2), was originally developed in our previous study for nurses in Vietnam [16]. This program can be accessed using smartphone apps downloadable from the Apple App Store and Google Play Store as well as on personal computers and other devices connected to the internet. The original program [16] was developed in Vietnamese by researchers from Japan and Vietnam. Appropriate cultural adaptations were safeguarded through an extensive discussion among researchers and a group of senior nurses. A case story that would be used in the program to illustrate the stress process and stress management was developed, considering popular job stressors in the target group, such as job overload, role conflict, and work-family interference. The cartoon character images of a counselor and a nurse were carefully created to be appropriate for the setting. These adaptations were assessed to be appropriate for Thailand’s culture and setting. The COVID-19 module was developed through a discussion among researchers from 3 countries; it was accommodated and adapted to the cultures and COVID-19 epidemic situations of Vietnam and Thailand. The program comprised 7 modules that were presented in a fixed order. New modules were made available each week, starting from modules 1 to 7. The modules were based on 2 already established CBT packages: cognitive therapy by Beck [26] and coping with depression by Lewinsohn et al [27]. The modules focused on the following themes: a transactional model of stress and coping (module 1), a self-case formulation based on the cognitive behavioral model (module 2), behavioral activation (module 3), cognitive restructuring (module 4), cognitive restructuring and relaxation (module 5), problem-solving (module 6), and stress and coping for better mental health during the COVID-19 pandemic (module 7). Modules 1-6 had been developed using the modules of the previous iCBT program that had successfully reduced depression, as seen at the 3-month follow-up among nurses in Vietnam [16]. Each module required 5-10 minutes to read, without any exercise or quiz being offered. In the last part of each module, 4-panel comics were provided to engage the participants in the programs. The seventh module was developed afresh to specifically teach awareness of stress and coping with stress during the COVID-19 pandemic, based on a literature review [28-30]. Through a discussion by researchers from Japan, Vietnam, and Thailand, a draft was developed, which consisted of 6 sections [28-30]: (1) job stressors and mental health symptoms of nurses during the COVID-19 pandemic, (2) keeping a healthy lifestyle, (3) communicating with others, (4) applying stress management techniques, (5) seeking professional help if needed, and (6) coping with discrimination and bullying to health care professionals due to COVID-19. Most importantly, the fourth section was designed to introduce how participants could use the CBT skills that they learned in the earlier modules during the COVID-19 pandemic. This draft was reviewed and approved by research teams, including nurses from Vietnam and Thailand, considering the local situations and experiences of nurses during the COVID-19 pandemic. In Vietnam, a smartphone app was prepared and used by the participants to access the program. In Thailand, the participants were asked to access the program using a URL leading to the web program server. Some of the participants in Vietnam who could not install the app used a web-based version. We allowed participants to access the intervention program for an additional 3 weeks to allow those who did not complete all the modules during the initial 7 weeks to study the remaining modules. To facilitate the use of the program, the researchers sent a notice for opening a new module and a reminder to complete an unfinished module to the participants every week during the first 7 weeks of the intervention period in both countries. In Vietnam, the messages were sent via group chat, SMS text messages, and hotline support. In addition, head nurses in the hospitals also reminded the participants through group chats and meetings. In Thailand, the messages were sent only from the researchers via emails, texts, and chat apps individually.

#### Control as Treatment-As-Usual

The participants allocated to the control group did not receive any intervention program for the first 6 months. Participants in both the intervention and control groups were able to use an internal regular occupational health service at the targeted hospitals. They also could use employee assistance program services if the hospitals offered them. Further, they had access to a mental health care service in their community. The participants in the control group were provided a chance to use the iCBT program that was offered to the intervention group after the 6-month follow-up survey.

### Outcomes

#### Choice of Primary Measure

The primary outcome was the depressive symptoms that were measured by the Depression Anxiety Stress Scale-21 items (DASS21). The DASS21 was administered both in the intervention and control groups at baseline and at 3- and 6-month follow-ups. The DASS21 is a screening tool to assess depression, anxiety, and stress in the preceding 7 days [31]. It is free to use and is widely used for measuring depressive symptoms as the indicator in RCTs among nonclinical populations, including Asian countries. Vietnamese and Thai versions of DASS21 were developed and tested, and their internal consistency (Cronbach α) and validity were confirmed in previous studies [32,33]. In the sample of this study, Cronbach α coefficients of the scale were .83 in Vietnam and .86 in Thailand. The depression subscale comprises 7 items measuring dysphoria, hopelessness, and devaluation of life, among others. Each item is scored on a 4-point scale ranging from 0 (did not apply to me at all) to 3 (applied to me very much, or most of the time). The DASS21 depression score can range from 0 to 42, with higher scores indicating more severe depression; a cut-off score of 10 indicates mild depression [31]. The cut-off scores of moderate, severe, and extremely severe depression are 14, 21, and 28, respectively. Therefore, the 7-point change in DASS21 is considered a clinically significant change.

#### Process Evaluation

For the process evaluation, the completion rate of each module of the intervention program was collected from the digital records of the server system. A completion rate was calculated as the proportion of the participants who accessed, read, and worked on all the contents of the module until the close of the intervention program (10 weeks from the baseline survey).

### Sample Size Calculation

The sample size that was necessary to detect the effects of the intervention on reducing depression was calculated. According to a previous meta-analysis, a web-based universal prevention psychological intervention on reducing depression and anxiety in the workplace yielded a summary effect size of 0.25 [34]. Our study of an unguided universal prevention iCBT program among nurses in Vietnam reported a small effect size on depression (*d*=0.18 at the 3-month follow-up) [16]. To detect a minimal effect size of 0.15 at an α of .05 and power of .80, the estimated sample size was 699 participants in each group. A total of 1398 nurses participated in the study, combining samples from both countries. The statistical power was calculated using the G*Power version 3.1.9.2 [35].

### Randomization and Masking

The participants who met the inclusion criteria were randomly allocated to the intervention or control group. The randomization was stratified into 2×6×2 = 24 strata, based on country (Vietnam or Thailand), 6 hospitals, and subgroups based on the cutoff point of mild depression by the DASS21 scores at baseline (10 or greater and less than 10). The stratified permuted block randomization method was used, with a block size of 4. An independent biostatistician generated a password-protected stratified random table using SAS software (SAS Institute Inc). An independent research assistant at the Department of Mental Health, The University of Tokyo, conducted the assignment using the random table, which was blinded to the researchers. Because this trial offered a psychological intervention, it was impossible to blind the results of the allocation to the participants and researchers.

### Statistical Analysis

For the main analysis, mixed modeling for repeated measures was used to test the effects of the intervention compared with the control group at 3- and 6-month follow-ups. The interaction effect of the group (1=intervention, 0=control) by time was considered the indicator of the intervention effect. Variables used in the stratified randomization were entered as covariates: countries, hospitals, and subgroups based on the cutoff point of mild depression by DASS21. Intention-to-treat analysis was adopted, which included all the participants in the analysis who completed the baseline survey. Effect sizes were calculated as the standardized mean differences of the score in the DASS21 based on the estimated mean differences at the 3- and 6-month follow-ups. The effectiveness of the intervention was tested in subgroups of nurses in Vietnam and Thailand. There were no missing values during the baseline survey because we asked participants to respond to all the questions by using a web-based survey system. Missing values for DASS21 scores due to attrition were imputed by applying the maximum likelihood estimation of the MIXED procedure of SPSS Statistics (IBM Corp).

We conducted a post hoc subgroup analysis to test the prevention effect of the intervention by examining the difference in the incidences of mild depression (ie, ≥10 on the DASS21 score) among participants with no depression (ie, <10 on the DASS21 score) at the baseline between the intervention and control groups. We applied a logistic regression analysis to estimate the odds ratio of mild depression for the intervention group compared with the control group at the 3- and 6-month follow-ups. Missing values for DASS21 scores due to attrition were imputed by multiple imputations using MULTIPLE IMPUTATION procedure of SPSS statistics. A total of 50 pseudocomplete data sets were generated by the Markov chain Monte Carlo methods using regression models. We referred to pooled estimates of the 50 results. All statistical analyses were conducted using SPSS Statistics (version 29.0; IBM Corp).

### Change to the Protocol

The above post hoc subgroup analysis on the incidence of mild depression was conducted to clarify the pure prevention effect of the intervention, responding to a reviewer’s request, which was not initially described in the registered protocol [23].

## Results

### Participant Recruitment

**Figure 1:**
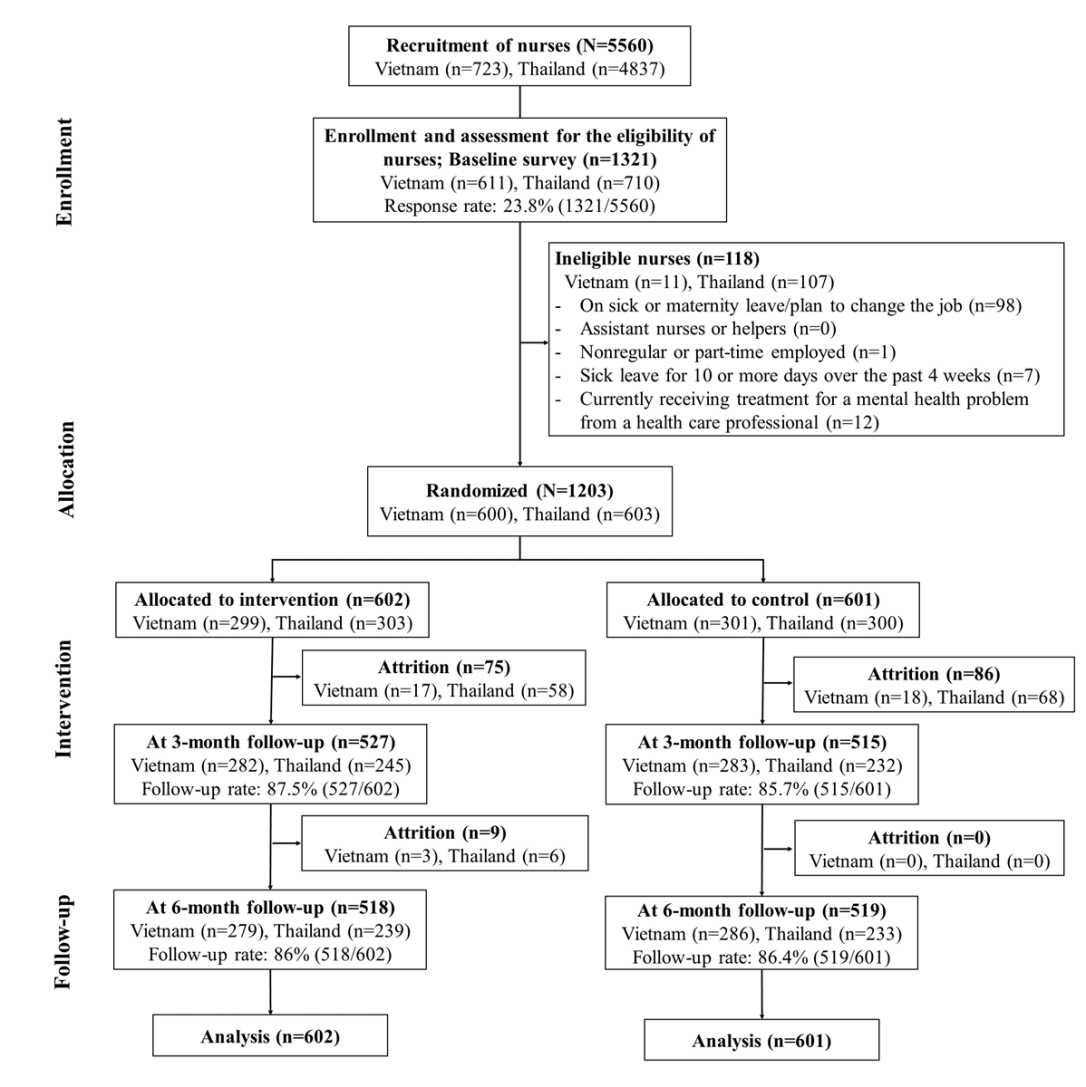
Flowchart of participant recruitment and allocation.

Recruitment and the baseline survey were conducted from March to April 2022 in Thailand and Vietnam. Among the 5560 recruited nurses, 1321 (23.8%) completed the baseline survey. After screening for eligibility, 118 nurses were considered ineligible and excluded. Most ineligible nurses were those who were/planned to go on sick or maternity leave or planned to change jobs (n=98). Following the stratified randomization of the included nurses (N=1203), 602 nurses were allocated to the intervention group and 601 nurses to the control group. The 3- and 6-month follow-up surveys were conducted from July to August 2022 and from October to November 2022, respectively. At the 3-month follow-up, 87.5% (527/602) of the nurses and 85.7% (515/601) of the nurses in the intervention and control groups, respectively, completed the follow-up survey. At the 6-month follow-up, 86% (518/602) of the nurses and 86.4% (519/601) of the nurses in the intervention and control groups, respectively, completed the survey (Figure 1).

### Baseline Characteristics

At baseline, 88.1% (1060/1203) of the participants were females, and 60.9% (733/1203) of them had a spouse (Table 1). Almost all participants (1162/1203) had obtained a college or university degree, and 83.4% (1004/1203) had no-term contracts with hospitals. About 22.6% (272/1203) of the participants indicated having mild or severe depression at baseline. Demographic characteristics were not very different between the 2 groups. Statistical tests (*χ*^2^ tests and 2-sided *t* tests) indicated that the demographic differences were not significant (*P* for differences ranged from .17 to .87).

**Table 1:** Characteristics of the participants at baseline.

		Intervention group				Control group				*P* value (difference)	
		Vietnam (n=299)	Thailand (n=303)	Total (n=602)	Vietnam (n=301)		Thailand (n=300)	Total (n=601)			
**Hospital, n (%)**											N/A^a^
	Vietnam 1	100 (33.4)	N/A	100 (16.6)	100 (33.2)		N/A	100 (16.6)			
	Vietnam 2	199 (66.6)	N/A	199 (33.1)	201 (66.8)		N/A	201 (33.4)			
	Thailand 1	N/A	78 (25.7)	78 (13)	N/A		75 (25)	75 (12.5)			
	Thailand 2	N/A	79 (26.1)	79 (13.1)	N/A		78 (26)	78 (13)			
	Thailand 3	N/A	88 (29)	88 (14.6)	N/A		87 (29)	87 (14.5)			
	Thailand 4	N/A	58 (19.1)	58 (9.6)	N/A		60 (20)	60 (10)			
**Employment contract, n (%)**											.87
	Fixed-term contract	27 (9)	59 (19.5)	86 (14.3)	26 (8.6)		57 (19)	83 (13.8)			
	No-term contract	267 (89.3)	238 (78.5)	505 (83.9)	260 (86.4)		239 (79.7)	499 (83)			
	Others	2 (0.7)	6 (2)	8 (1.3)	2 (0.7)		4 (1.3)	6 (1)			
	Missing	3 (1)	0 (0)	3 (0.5)	13 (4.3)		0 (0)	13 (2.2)			
**Gender, n (%)**											.27
	Male	60 (20.1)	6 (2)	66 (11)	68 (22.6)		7 (2.3)	75 (12.5)			
	Female	239 (79.9)	295 (97.4)	534 (88.7)	233 (77.4)		293 (97.7)	526 (87.5)			
	Others	0 (0)	2 (0.7)	2 (0.3)	0 (0)		0 (0)	0 (0)			
**Age (years), mean (SD)**		36.70 (7.7)	37.19 (10.7)	36.95 (9.3)	36.15 (6.9)		36.69 (9.9)	36.42 (8.5)	.31		
	Missing, n (%)	0 (0)	0 (0)	0 (0)	1 (0.3)		0 (0)	1 (0.2)			
**Marital status, n (%)**											.83
	Unmarried	40 (13.4)	173 (57.1)	213 (35.4)	45 (15)		173 (57.7)	218 (36.3)			
	Have a spouse	248 (82.9)	122 (40.3)	370 (61.5)	251 (83.4)		112 (37.3)	363 (60.4)			
	Separated/divorced/widowed	9 (3)	8 (2.6)	17 (2.8)	5 (1.7)		15 (5)	20 (3.3)			
	Missing	2 (0.7)	0 (0)	2 (0.3)	0 (0)		0 (0)	0 (0)			
**Highest educational qualification, n (%)**											.31
	Intermediate	25 (8.4)	0 (0)	25 (4.2)	14 (4.7)		0 (0)	14 (2.3)			
	College	150 (50.2)	0 (0)	150 (24.9)	149 (49.5)		0 (0)	149 (24.8)			
	University	117 (39.1)	252 (83.2)	369 (61.3)	124 (41.2)		259 (86.3)	383 (63.7)			
	Postgraduate	7 (2.3)	51 (16.8)	58 (9.6)	12 (4)		41 (13.7)	53 (8.8)			
	Missing	0 (0)	0 (0)	0 (0)	2 (0.7)		0 (0)	2 (0.3)			
**Income in Vietnam (per month), n (%)^b^**											.40
	<₫2.1 million	3 (1)	N/A	3 (0.5)	1 (0.3)		N/A	1 (0.2)			
	₫2.1 to <₫4.2 million	15 (5)	N/A	15 (2.5)	14 (4.7)		N/A	14 (2.3)			
	₫4.2 to <₫6.3 million	145 (48.5)	N/A	145 (24.1)	129 (42.9)		N/A	129 (21.5)			
	₫6.3 to <₫8.4 million	77 (25.8)	N/A	77 (12.8)	96 (31.9)		N/A	96 (16)			
	≥₫8.4 million	59 (19.7)	N/A	59 (9.8)	58 (19.3)		N/A	58 (9.7)			
**Income in Thailand (per month), n (%)^c^**											.17
	<THB 20,000	N/A	2 (0.7)	2 (0.3)	N/A		9 (3)	9 (1.5)			
	THB 20,000 to <THB 40,000	N/A	159 (52.5)	159 (26.4)	N/A		159 (53)	159 (26.5)			
	THB 40,000 to <THB 60,000	N/A	107 (35.3)	107 (17.8)	N/A		107 (35.7)	107 (17.8)			
	THB 60,000 to <THB 80,000	N/A	30 (9.9)	30 (5)	N/A		20 (6.7)	20 (3.3)			
	≥THB 80,000	N/A	5 (1.7)	5 (0.8)	N/A		5 (1.7)	5 (0.8)			
	Missing	0 (0)	0 (0)	0 (0)	3 (1)		0 (0)	3 (0.5)			
**DASS21^d^ depression subscale score, n (%)**											.59
	No depression (<10 on the scale)	243 (81.3)	219 (72.3)	462 (76.7)	243 (80.7)		226 (75.3)	469 (78)			
	Mild depression (≥10 on the scale)	56 (18.7)	84 (27.7)	140 (23.3)	58 (19.3)		74 (24.7)	132 (22)			

^a^N/A: not applicable.

^b^As of March 2022, US $1=₫22,830.

^c^As of March 2022, US $1=THB 33.

^d^DASS21: Depression Anxiety Stress Scale-21 items.

### Effects of the Intervention Program on Depression

In both groups, the average scores of depression at the 3- and 6-month follow-ups were lower than those at the baseline (Table 2). The scores in the intervention group decreased steeply at the 3-month follow-up and returned slightly to the baseline scores. The scores in the control group decreased monotonically. Cohen *d* scores calculated from the descriptive statistics were –0.13 (95% CI –0.26 to –0.02) at the 3-month follow-up and –0.05 (95% CI –0.17 to –0.02) at the 6-month follow-up. For the country subgroups, Cohen *d* scores at the 3-month follow-up were almost the same in Thailand (*d*=–0.15, 95% CI –0.33 to 0.03) and Vietnam (*d*=–0.13, 95% CI –0.31 to 0.03), while the scores at the 6-month follow-up in Thailand (*d*=–0.12, 95% CI –0.31 to 0.06) were greater than those in Vietnam (*d*=0.02, 95% CI –0.14 to 0.19). The number of missing values for depression due to attrition was not significantly different between the intervention and the control groups (*P*=.82). Compared with those who were survey completers, the participants who had fixed-term contracts (*P*<.001), were young (*P*=.02), were unmarried (*P*<.001), and had postgraduate qualifications (*P*<.001) tended to drop out from the follow-up surveys. The difference in the rate of mild or severe depression at baseline was not significant (*P*=.10).

**Table 2:** Mean (SD) of Depression Anxiety and Stress Scale-21 items depression subscale scores at baseline and at the 3- and 6-month follow-ups.

	Intervention group (n=602)					Control group (n=601)					Effect size (intervention vs control), Cohen *d* (95% CI)		
	V^a^, mean (SD)	T^b^, mean (SD)	Total, mean (SD)	Missing, n (%)	V, mean (SD)		T, mean (SD)	Total, mean (SD)	Missing, n (%)	V		T	Total
Baseline	5.05 (5.6)	6.27 (6.1)	5.66 (5.9)	N/A^c^	4.92 (5.3)		6.40 (6.5)	5.66 (6.0)	N/A	N/A		N/A	N/A
3-month follow-up	4.07 (4.8)	3.40 (5.3)	3.75 (5.0)	101 (16.8)	4.69 (5.4)		4.46 (6.4)	4.58 (5.9)	80 (13.3)	–0.13 (–0.31 to 0.03)		–0.15 (–0.33 to 0.03)	–0.13 (–0.26 to –0.02)
6-month follow-up	4.75 (6.2)	3.04 (4.8)	3.95 (5.6)	86 (14.3)	4.49 (6.2)		3.96 (6.1)	4.25 (6.1)	89 (14.8)	0.02 (–0.14 to 0.19)		–0.12 (–0.31 to 0.06)	–0.05 (–0.17 to 0.07)

^a^V: Vietnam.

^b^T: Thailand.

^c^N/A: not applicable.

Following mixed modeling (Table 3), the group difference in the score change was significant and nonsignificant at the 3-month (coefficient=–0.92, 95% CI –1.66 to –0.18; *P*=.02) and 6-month follow-ups (coefficient=–0.33, 95% CI –1.11 to 0.45; *P*=.41), respectively. The estimated effect sizes were –0.15 and –0.06 at the 3- and 6-month follow-ups, respectively. For the country subgroups, a significant group difference was observed at the 3-month follow-up in Thailand (coefficient=–1.24, 95% CI –2.26 to –0.23; *P*=.02) and about twice as greater than that in Vietnam (coefficient=–0.66, 95% CI –1.70 to 0.39; *P*=.22). The estimated effect size at the 3-month follow-up in Thailand was –0.21. The coefficients at the 6-month follow-up were nonsignificant in either country; the direction of the effect was maintained in Thailand (coefficient=–0.83, 95% CI –1.78 to 0.12; *P*=.09) but reversed in Vietnam (coefficient=0.12, 95% CI –1.04 to 1.29; *P*=.84).

**Table 3:** Effects of the intervention program on depression.

			Coefficient^a^ (95% CI)		SE		*t* test *(df)*		*P* value		Estimated effect size (95% CI)^b^
**Total sample** **(N=1203)**											
	3-month follow-up	–0.92 (–1.66 to –0.18)		0.38		–2.44 (1331.34)		.02		–0.15 (–0.28 to –0.03)	
	6-month follow-up	–0.33 (–1.11 to 0.45)		0.40		–0.83 (1296.85)		.41		–0.06 (–0.19 to 0.08)	
	Pooled^c^	–0.06 (–0.19 to 0.08)		0.07		–0.85 (1196.36)		.39		–0.01 (–0.03 to 0.01)	
**Vietnam** **(n=600)**											
	3-month follow-up	–0.66 (–1.70 to 0.39)		0.53		–1.23 (709.20)		.22		–0.11 (–0.29 to 0.07)	
	6-month follow-up	0.12 (–1.04 to1.29)		0.60		0.21 (696.80)		.84		0.02 (–0.18 to 0.22)	
	Pooled	0.14 (–0.06 to 0.34)		0.10		0.14 (650.99)		.89		0.02 (–0.01 to 0.06)	
**Thailand** **(n=603)**											
	3-month follow-up	–1.24 (–2.26 to –0.23)		0.52		–2.40 (636.48)		.02		–0.21 (–0.38 to –0.04)	
	6-month follow-up	–0.83 (–1.78 to 0.12)		0.49		–1.70 (608.97)		.09		–0.14 (–0.30 to 0.02)	
	Pooled	–0.13 (–0.30 to 0.03)		0.08		–1.60 (541.01)		.11		–0.02 (–0.05 to 0.01)	

^a^Coefficients were adjusted by the variables used in the stratified randomization: country (Vietnam and Thailand), hospital (6 hospitals), and severity of depression (≥10 or <10 on the Depression Anxiety Stress Scale-21 items depression subscale).

^b^Effect sizes were calculated by dividing the estimated effect by the pooled SD.

^c^Mixed models for repeated measures conditional growth model analyses were conducted.

Among the participants with no depression at the baseline (n=931), 85 (9.1%) reported mild depression at the 3-month follow-up. The proportions of new cases of mild depression were 7.8% (36/462) in the intervention group and 10.4% (49/469) in the control group at the 3-month follow-up. For the result of logistic regression, the pooled estimate of odds ratio for mild depression of the intervention group compared with the control group was 0.70 (95% CI 0.45-1.10; *P*=.12). At the 6-month follow-up, 88 (9.5%) reported mild depression. Proportions of mild depression were 8.9% (41/462) in the intervention group and 10% (47/469) in the control group. The pooled estimate of odds ratio was 0.94 (95% CI 0.61-1.45; *P*=.79).

### Process Evaluation

The completion rates in each module ranged from 87.5% (527/602) to 74.3% (447/602) and decreased monotonically from modules 1 to 7 (Table 4). Approximately 68.1% (410/602) of the participants completed all the 7 modules. The completion rates of modules 1 to 5 in Vietnam were significantly higher than those in Thailand (Table 4). There were no significant differences in the rates for modules 6 and 7 as well as the proportions of completers of all the 7 modules between Vietnam and Thailand.

**Table 4:** Completion rates for each module and all 7 modules.

			Vietnam (n=299), n (%)		Thailand (n=303), n (%)		Total (n=602), n (%)		Difference in 2 countries, *χ*^2^ (*df*)			*P* value	
**Module 1**										9.1 (1)			.003
	Complete	274 (91.6)		253 (83.5)		527 (87.5)							
	Incomplete	25 (8.4)		50 (16.5)		75 (12.5)							
**Module 2**										11.9 (1)			<.001
	Complete	272 (91)		246 (81.2)		518 (86)							
	Incomplete	27 (9)		57 (18.8)		84 (14)							
**Module 3**										12.2 (1)			<.001
	Complete	270 (90.3)		243 (80.2)		513 (85.2)							
	Incomplete	29 (9.7)		60 (19.8)		89 (14.8)							
**Module 4**										6.6 (1)			.01
	Complete	262 (87.6)		242 (79.9)		504 (83.7)							
	Incomplete	37 (12.4)		61 (20.1)		98 (16.3)							
**Module 5**										5.7 (1)			.02
	Complete	258 (86.3)		239 (78.9)		497 (82.6)							
	Incomplete	41 (13.7)		64 (21.1)		105 (17.4)							
**Module 6**										0.3 (1)			.57
	Complete	225 (75.3)		234 (77.2)		459 (76.2)							
	Incomplete	74 (24.7)		69 (22.8)		143 (23.8)							
**Module 7**										3.5 (1)			.06
	Complete	212 (70.9)		235 (77.6)		447 (74.3)							
	Incomplete	87 (29.1)		68 (22.4)		155 (25.7)							
**All 7 modules**										1.8 (1)			.18
	Complete	196 (65.6)		214 (70.6)		410 (68.1)							
	Incomplete	103 (34.4)		89 (29.4)		192 (31.9)							

## Discussion

### Principal Results

The findings of our multi-country RCT show that the smartphone-based self-guided iCBT program, “The ABC Stress Management–COVID-19 version,” significantly reduced depression at the 3-month follow-up among hospital nurses in Vietnam and Thailand. However, the estimated effect size was small (–0.15), and therefore, these results may not be clinically meaningful. Furthermore, the effect of the program leveled off and was nonsignificant at the 6-month follow-up. The incidence of mild depression was reduced by 30% among participants with no depression at the baseline in the intervention group at the 3-month follow-up, but this prevention effect was not statistically significant. The effect of the program in Thailand was slightly greater than that in Vietnam, while the pattern of change in the depression scores was similar. About two-third or more of the participants completed all the 7 modules during the intervention period.

This is the first study, to the best of our knowledge, that has demonstrated the effect of a smartphone-based iCBT universal program for prevention of depression among nurses during the COVID-19 pandemic. Our findings are consistent with those of a previous RCT on the effectiveness of an internet-based program for psychological distress among non–health care workers during the COVID-19 pandemic [36]. A self-guided smartphone-based stress management program may be effective for reducing depression among hospital nurses during the COVID-19 pandemic. A factor for this positive finding could be the high completion rate of the program: the process evaluation revealed that more than 80% of the participants in the intervention group completed the minimum intervention (module 1) and more than two-thirds completed all the modules. This was possibly due to the intensive follow-up procedures that we used during the intervention period. In a similar previous study [19], which failed to show the effect of an internet stress management program, there might not have been enough high intensity for the follow-up to achieve a significant finding on the effectiveness; however, the completion rate was not reported.

The effect of our program attenuated at the 6-month follow-up. A similar pattern was observed in our previous RCT using a similar self-guided smartphone-based stress management program among hospital nurses in Vietnam before the COVID-19 pandemic [16]. When the intervention is nonintensive such as a self-guided program, a web-based psychosocial intervention may be effective for a short period but not for a longer period [37]. However, a group-based psychological first aid training program was reported to improve professional efficacy at the 6-month follow-up among health care workers during an Ebola outbreak in Africa [4]. The attenuation of the effect might be attributable to the low-intensity, self-guided nature of the program. Probably, the participants were not able to retain their learnings from the program in their memory for a longer duration. The other possibility is that the overwhelming stressful experiences that they may have had due to the COVID-19 pandemic could have limited them from using coping strategies learned from the program for a longer period [38]. One study showed that a group-based psychological intervention for the mental health of mothers after a nuclear disaster was effective for 1 month but not for 3 months [39]. Therefore, the severity of a disaster experience might diminish the long-term effect of a stress management program. This should be investigated further by, for instance, looking at the moderating effect of disaster-related (ie, COVID-19–related in this context) stressful experiences during a postintervention follow-up on the effect of a stress management program.

Although the effect of our program on depression was similar in Vietnam and Thailand, the effect in Thailand was slightly larger than that in Vietnam. This may be attributed to the differences in the sampling process: in Vietnam, all the nurses from the collaborating target hospitals were potential participants, while in Thailand, selected nurses from target hospitals were recruited for this study. The participants in Thailand may have included those who were more motivated to participate in the program, thereby enhancing the effect of the program. The other possibility could be the different timings and patterns of COVID-19 outbreaks and related responsiveness between Vietnam and Thailand. At the country level, the total number of confirmed cases in Vietnam was much more than that in Thailand in 2022, although the peaks of the outbreak were similar around March 2022 [21]. It is possible that COVID-19 hit the target hospitals in Vietnam more severely than that in Thailand, and participants in Vietnam may have been more overwhelmed by the situation and therefore less able to cope using what they learned in the program. The completion rates of all 7 modules by nurses in Thailand were greater than those by nurses in Vietnam; in particular, higher proportions of participants in Thailand studied module 7 (coping with COVID-19 stress), while the completion rates for modules 1 to 5 were higher in Vietnam. Module 7 (stress and coping for better mental health during the COVID-19 pandemic) completion rate was higher in Thailand. Module 7 might suit the participants’ stressful situations during the COVID-19 pandemic period and lead to higher effectiveness among nurses in Thailand. This, in turn, may have led to slightly better findings among nurses in Thailand. A module connecting CBT skills with stress in the COVID-19 pandemic may assist participants in understanding how to cope with stress in the COVID-19 situation.

### Limitations

This study had several limitations. The follow-up rates were relatively high at 3- and 6-month follow-ups in both groups. However, there was a possible attrition bias that may affect the findings. If the less motivated participants dropped out, the effects of the program would be overestimated. Because the iCBT program was a psychosocial intervention, it was impossible to blind the participants to the group. The intervention group could have been more motivated to report their depression as less than the exact level at the follow-ups. If the participants in the intervention group shared the information about the program content with the participants in the control group, the observed effect might have been underestimated. Generalization of the findings may also be limited. Our findings were obtained from nurses from a limited number of large provincial hospitals and may not apply to other countries, including lower or higher-income countries and countries in other regions and with different cultures. In addition, because the participants in Thailand were volunteers who were motivated to complete the program, the findings may not be generalizable to the entire population of nurses at the target hospitals.

### Conclusions

Our study demonstrates that the smartphone-based iCBT program was effective in improving depression at the 3-month follow-up among hospital nurses in Vietnam and Thailand during the COVID-19 pandemic. This program was self-guided, not requiring support from professional therapists. This program did not need face-to-face contact to study the content. Thus, it could be disseminated to a large population of hospital nurses in low-resource settings during the outbreak crisis, with social distancing restrictions and reduced risk of infection. In addition, this program is suitable for health care workers who work irregular schedules because it can be completed at their convenient time. This program may be beneficial for the mental health and well-being of nurses who face stress due to the COVID-19 pandemic in these countries. It may also contribute to developing a psychologically resilient nursing workforce during such challenging times, which would help maintain health care services to patients. This program should be evaluated for its effectiveness in other middle-income countries in Southeast Asia and during other infectious disease outbreaks. Nonetheless, it is a promising approach for reducing the depression of nurses in a future infectious disease pandemic.

## Appendix

Multimedia Appendix 1 CONSORT-eHEALTH checklist (V 1.6.1).

Multimedia Appendix 2 Screenshot of The ABC Stress Management–COVID-19 version.

## Abbreviations

COCONATS: Coping with COVID-19 helps Nurses be Active, Tough, and Smiling
CONSORT: Consolidated Standards of Reporting Trials
DASS21: Depression Anxiety Stress Scale-21 items
iCBT: internet-based cognitive behavioral therapy
RCT: randomized controlled trial
