# Supplementary figures and images for: Effectiveness of a Smartphone-Based Stress Management Program for Depression in Hospital Nurses During COVID-19 in Vietnam and Thailand: 2-Arm Parallel-Group Randomized Controlled Trial
Source: J Med Internet Res. 2024 Aug 30;26:e50071. doi: 10.2196/50071 (PMC11399744; doi:10.2196/50071)

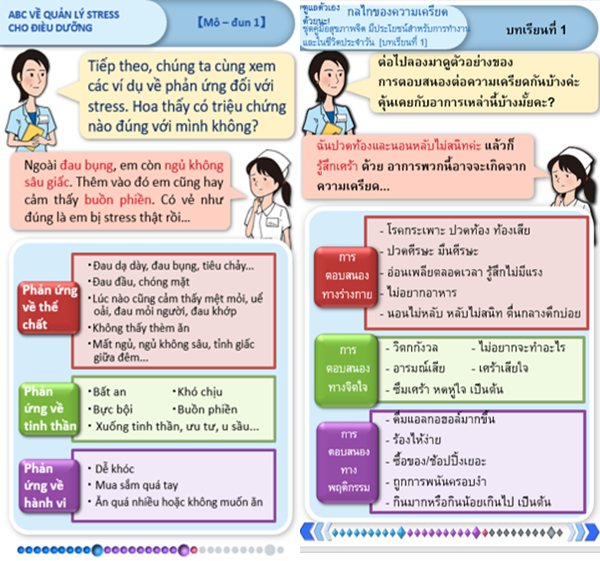

Supplement: Multimedia Appendix 2 [file jmir_v26i1e50071_app2.png]
